# Simulating the computational mechanisms of cognitive and behavioral psychotherapeutic interventions: insights from active inference

**DOI:** 10.1038/s41598-021-89047-0

**Published:** 2021-05-12

**Authors:** Ryan Smith, Michael Moutoussis, Edda Bilek

**Affiliations:** 1 Laureate Institute for Brain Research, 6655 S Yale Ave, Tulsa, OK 74136 USA; 2 Wellcome Centre for Human Neuroimaging, Institute of Neurology, University College London, London, UK; 3 The Max Planck—University College London Centre for Computational Psychiatry and Ageing, London, UK

**Keywords:** Computational neuroscience, Psychology

## Abstract

Cognitive-behavioral therapy (CBT) leverages interactions between thoughts, feelings, and behaviors. To deepen understanding of these interactions, we present a computational (active inference) model of CBT that allows formal simulations of interactions between cognitive interventions (i.e., cognitive restructuring) and behavioral interventions (i.e., exposure) in producing adaptive behavior change (i.e., reducing maladaptive avoidance behavior). Using spider phobia as a concrete example of maladaptive avoidance more generally, we show simulations indicating that when conscious beliefs about safety/danger have strong interactions with affective/behavioral outcomes, behavioral change during exposure therapy is mediated by changes in these beliefs, preventing generalization. In contrast, when these interactions are weakened, and cognitive restructuring only induces belief uncertainty (as opposed to strong safety beliefs), behavior change leads to generalized learning (i.e., “over-writing” the implicit beliefs about action-outcome mappings that directly produce avoidance). The individual is therefore equipped to face any new context, safe or dangerous, remaining in a content state without the need for avoidance behavior—increasing resilience from a CBT perspective. These results show how the same changes in behavior during CBT can be due to distinct underlying mechanisms; they predict lower rates of relapse when cognitive interventions focus on inducing uncertainty and on reducing the effects of automatic negative thoughts on behavior.

## Introduction

‘*I do not understand what I do. For what I want to do I do not do, but what I hate I do. … I have the desire to do what is good, but I cannot carry it out.*’ Paul, 56 ACE.

**Figure 1:**
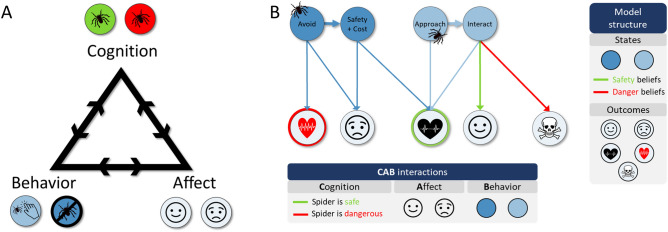
Illustration of how the structure of the computational model described in this paper captures the cognition-affect-behavior triangle, using the example of spider phobia. (**A**) Cognition-affect-behavior triangle, as used in CBT. Each component has reciprocal links to the other two components. For example, a thought can promote congruent emotions and behaviors, and an emotion can also promote congruent thoughts and behaviors. In this case, the thought “the spider is dangerous” may promote “negative affect’ and “running away” (circles on the right-hand side of each apex). (**B**) Simplified depiction of the computational model structure, designed to capture the outcomes an individual expects if they choose approach or avoidance behavior after seeing a feared stimulus (a spider in this example), depending on their thoughts (here, beliefs that a spider is dangerous or safe). Cognition and behavior within the CBT triangle form the model states. Affect and arousal are outcomes, which depend on cognitions and chosen behaviors, as well as their interactions. This figure includes icons created by the Noun Project (https://thenounproject.com/).

This paper seeks to use computational modelling to deepen our understanding of how thoughts interact with defensive but maladaptive actions in common mental disorders, an issue very important for cognitive-behavioral therapy (CBT^1,2^). CBT is one of the leading mental health interventions for such disorders, yet many patients are not helped by it (e.g., see^3^). According to CBT, patients engage in behaviors that are ultimately unhelpful or maladaptive, though in the short term they help reduce distress. A key way in which CBT helps patients overcome such problems is *exposure*, during which patients stay with a feared/avoided situation until arousal declines and/or they learn by experience that arousal and anxiety abate and no adverse outcomes ensue (or that adverse outcomes are tolerable)—leading both internal and external stimuli to lose their sting^4^. CBT also addresses the unhelpful or distorted thoughts in a patient’s problem situations, as it holds that these thoughts can lead to maladaptive actions and greatly contribute to affective distress. Thus, thoughts, affect, and behavior form a closely interacting triangle, where each element affects each of the others (e.g., the thought “this is dangerous” leads to fearful affect and actions that seek safety). This *cognitive triangle* constitutes one of the fundamental concepts of CBT (Fig. 1A illustrates this in the example case of spider phobia^1^). During therapy, patients learn to differentiate the three components of this triangle for any given experience. A beneficial change in one component can then be observed to affect the others (e.g., trying a new behavior and observing the resulting affect and related thoughts). Importantly, the degree to which each component of the triangle influences the other two varies across individuals and contexts—a consideration highly relevant to treatment selection^5^. It is a common clinical observation that directly targeting thoughts (e.g., through *cognitive restructuring* interventions in CBT; see Table 1) can be highly effective at changing affect and behavior in some individuals, but less so in others.

**Table 1:** Terminology.

CBT terms	Description
Avoidance	Emission or withholding of activity so as not to enter a feared situation. For example, ‘school avoidance’ = not going to school
Aversion	Emotional state indicating strong dislike for a stimulus. This is often followed by congruent behaviors, such as avoidance
Catastrophizing	A cognitive bias to occupy oneself with (and expect) the worst possible outcome of a situation, while disregarding its (un-)likelihood. This may also include confident (but unwarranted) expectations of great discomfort, harm, or loss
Cognitive restructuring	The therapeutic process of identifying and altering maladaptive beliefs through a number of CBT interventions, such as Socratic dialogues, reappraisal, or reattribution
Dysfunctional assumption	Beliefs such as ‘unless I always succeed, I’m a loser’, used as a rule-guiding behavior (‘… so I must always succeed’). It can lead to distorted or otherwise unhelpful inference (‘I failed in this, what a loser I am!’) and maladaptive behavior
Exposure	Entering a feared situation. During CBT, exposure typically entails remaining in a feared situation until subjective fear has returned to adaptive levels while the patient withholds safety behaviors. See also ‘response prevention’
Psychotherapy	A formal treatment aiming to change cognition, perception, and behavior in order to improve mental health. Here we include at least the psychological therapies of all modalities (behavioral, analytic, systemic, etc.) and the rehabilitation therapies
Response prevention	Withholding safety behaviors (see below) upon entering a feared situation (i.e., what happens in Exposure with Response Prevention, or ERP)
Safety behavior	In general, any behavior carried out in order to regulate an emotional state or to avert a serious feared outcome. In this sense, avoidance is a safety behavior that removes the patient from an anxiogenic situation. However, ‘safety behavior’ usually means protective behaviors emitted within an anxiogenic situation

Active inference terms	Description
Hidden states	States of the world that are not directly observable and must be inferred from sensory input. In other words, these correspond to internal representations of the possible causes of directly observable outcomes. The most likely hidden states need to be inferred from observations
Bayesian beliefs	Probability distributions over hidden states of the world, which encode the strength of belief that one or another of those hidden states is the true state. Individuals need not be aware of these internally represented probability distributions, but could be in some cases. Put another way, these distributions could be identified with psychological thoughts or beliefs when modelling some phenomena, but less plausibly so when modelling other phenomena
Prior beliefs	Initial beliefs about states prior to making a new observation
Outcomes	Observations that are assumed to be caused by hidden states

Model-specific terms	Description
Explicit beliefs	Consciously accessible and verbally reportable beliefs about hidden states. The hidden state factor corresponding to explicit beliefs in the current model corresponds to safety/danger beliefs. These beliefs are probabilistic, such that an individual can assign different probabilities to (or have different levels of confidence in) the possibility that a spider is safe or dangerous (e.g., a belief that there is an 80% chance a spider is dangerous)
Implicit beliefs	Innate or learned associations mediating automatic reactions to a stimulus, which can in some cases contradict explicit (reportable) beliefs. These modulate behavior or conscious thoughts, but are not themselves directly accessible to the awareness of a patient. For example, in our model this could be an implicit association between approaching a spider and serious harm (even if a patient explicitly believes the spider is safe), which generates high arousal, negative affect, and avoidance motivation. To be clear, actual patients could also have conscious beliefs about their implicit expectations, but this would require an additional hidden state factor not included in our model. The implicit associative beliefs here can be thought of as those underlying conditioned and unconditioned responses

In order to clarify the mechanisms underlying such individual differences, in this paper we introduce a computational model affording simulations of cognitive-behavioral interventions and the associated cognitive-affective-behavioral (CAB) interactions described by the CBT triangle. Using the example of spider phobia, Fig. 1B illustrates the conceptual structure of this model, in which different behaviors lead to different affective states, arousal levels, and possible harm, depending on an individual’s beliefs about whether a spider is safe or dangerous. However, this abstract structure is not specific to spider phobia, and is meant to generalize to other cases of avoidance (e.g., agoraphobia, panic, social anxiety). This allows us to address specific research questions about the kinds of belief updating and learning mechanisms that may underlie reductions in avoidance behavior in response to CBT more generally, and if those mechanisms may differ in response to cognitive vs. behavioral interventions. The scope of the model is therefore focused on studying these mechanisms, as opposed to reproducing the complex and heterogeneous details of particular clinical cases. We aim to generate novel insights about approach-avoidance learning mechanisms that could generalize across multiple disorders and make empirical predictions. That said, in various places throughout the manuscript we will highlight how the model might be expanded or narrowed to focus on more specific case details.

One motivating observation is that people often act against their explicit beliefs, especially in the presence of strong feelings. This may be maladaptive, as the opening biblical quote suggests; but in CBT it can also be used to good effect. Here, patients may enter behavioral (esp., exposure-based) interventions with or without first adjusting their cognitions. In fear-based disorders, cognitive interventions may first establish explicit beliefs of safety, or may only generate uncertainty about the accuracy of previous anxiety-promoting beliefs. When anxiety-promoting, unhelpful or distorted thoughts persist, especially when they represent automatic and rigid evaluations, patients can learn to diagnose them as just that, thoughts. Thus, thoughts can be observed and do not have to be acted upon (this is also a core component of other evidence-based therapies, such as cognitive distancing within acceptance and commitment therapy [ACT]^6,7^). Consequently, patients can (1) exhibit adaptive behavior despite persistent and unhelpful automatic thoughts, (2) gain novel experiences as a result, and (3) adapt their behavior according to the observed consequences.

For many patients CBT is unhelpful, and much qualitative and empirical research has tried to address these modes of failure, but these problems regarding efficacy persist^8^. Our understanding of CAB interactions in cases of poor therapeutic efficacy remains incomplete. Here, novel computational psychiatry approaches may help, as they offer a mechanistic theory of the “flow” from perception, to elicitation of actions, to updating beliefs and the experience of affective states, such as feelings of unpleasant high arousal and anxiety^9–13^. As we will show, the CAB interactions described by the cognitive triangle in CBT emerge naturally out of computational models of the perception and decision-making problems faced by patients in problem contexts. Such models can offer explanations for why and how different therapeutic interventions result in specific arousal responses and behavioral outcomes for the individual (i.e., mechanistic explanations of individual differences in treatment response), enabling generalized behavioral improvements in some cases, but preventing generalization in others (for related computational work on this topic, see^14–16^). In what follows, we will now introduce the structure of our model and describe how it will be used to answer our questions of interest through simulation experiments.

## Methods

### An active inference model of approach-avoidance behavior in CBT

Before introducing our model, let us ensure that cognitive-behavioral terms are clearly related to our computational dialect. We will use the term *explicit belief*s to refer to the kinds of thoughts that we discuss with our patients—for example, “spiders are likely to bite me”, or “I am in danger”. These are explicit in the sense that, in our model, they are assumed to be consciously accessible to the patient and can be verbally articulated (conscious reporting has been modeled in detail elsewhere; e.g., see^17^). These types of beliefs would also be expected to be sensitive to reasoning and verbal instruction (although we do not explicitly model these aspects here). The model presented below more specifically casts these explicit thoughts/beliefs as probability distributions or *Bayesian beliefs*. In contrast, we will use the term *implicit beliefs* to refer to innate or learned associations between beliefs, actions, and their observable consequences. Implicit beliefs can influence thoughts and behavior, but are not themselves directly accessible to the awareness of a patient. For example, instructed extinction studies in anxiety research have shown that conditioned stimuli can in some cases continue to elicit unpleasant feelings, arousal and/or avoidance motivation, despite a participant being explicitly informed that the unconditioned (aversive) stimulus will no longer be presented^18^. In such cases the brain unconsciously ‘expects’ an aversive outcome (in an associative sense), despite an opposing conscious belief that no negative outcome will follow (although note that instructed extinction is also quite effective in other cases). In the present model, implicit beliefs are responsible for generating automatic increases in unpleasant arousal and avoidance motivation, which may or may not match with explicit beliefs (e.g., unpleasant arousal in response to seeing a spider at the zoo, despite consciously believing there is no actual danger).

To be clear, we expect that (at least in cases of high emotional awareness) actual patients may also have conscious/explicit beliefs about some of their implicit expectations (e.g., reporting “I believe my heartrate will go up if I see a spider and that I will feel anxious”), but this would require an additional hidden state factor not included in our model (for active inference models of emotional awareness and its additional benefits, see^11–13^). Thus, while a more complex model could accommodate potentially self-reportable beliefs about associatively learned expectations, in our model implicit beliefs would not be reportable. For the purposes of our model, one can assume that individuals with an explicit belief that a stimulus is dangerous (for example) would also self-report their conscious expectations about dangerous outcomes.

The way that explicit and implicit beliefs interact in our model is central to the results we present below. Implicit beliefs associate approach/avoidance behavior with outcomes (e.g., such as the associations learned in operant conditioning studies), where such outcomes might include arousal levels, pleasant/unpleasant feelings, and desired/feared events. In contrast, explicit beliefs modulate these implicit associations. For example, if an individual were told that a situation was safe, an implicit association with negative outcomes (and associated avoidance motivation) could be attenuated. However, there may be individual differences in the effectiveness of explicit beliefs at attenuating the influence of implicit associations on behavior—such as when individuals acknowledge that their avoidance behavior is irrational (i.e., when knowing something is safe), but continue to avoid nonetheless (a phenomenon that we model below).

Here we chose to use the active inference modelling framework, as it provides a unified approach for modelling perception, learning, and decision-making under the types of uncertainty central to approach-avoidance behavior and anxiety. This can be especially useful for examining the internal cognitive and emotional processes that underlie differential behavioral responses, but that can be difficult to study in behavioral experiments alone. In this modelling framework, the world (including the body) furnishes *observations* (or *observed outcomes*) to the mind. These observations (e.g., patterns of light hitting the retina) are caused by underlying *states* of the world (such as the presence of a spider). States are themselves not directly observable (referred to as “hidden”) and need to be inferred from observations (e.g., the brain must infer the presence of a spider based on visual input). Further, mappings between hidden states and observations can be probabilistic, such that new observations may only increase or decrease the subjective probability of one state vs. another. We can thus think of the mind as processing information in terms of beliefs about hidden states (e.g., see^19–21^). For example, in our model below the simulated patient (or decision-making “agent” more generally) must infer (i.e., perceive) the presence of a spider after making a “spider observation” (e.g., sensory input from something small, black, and furry). As described above, explicit hidden state beliefs (e.g., “I see a spider”) influence behavior via implicit associative beliefs about the specific combinations of outcomes that will be observed if certain hidden states of the world (also sometimes referred to as “hidden causes”) are present. To be clear, the true states of the world (e.g., the actual presence of a dangerous spider and approach behavior) interact to generate observed outcomes (referred to as the generative *process*), while the agent has beliefs about how states of the world generate outcomes (its generative *model*) that may or may not be correct. However, the agent has control over some of the true states of the world (i.e., its behavior) and the outcomes they generate in conjunction with the states of the world it does not control (e.g., whether the spider is dangerous or not, the resulting valence/arousal response, etc.). In this case, for example, the explicit beliefs “I see a spider” and “spiders are dangerous” can jointly convey the implicit expectation that one will observe sensations associated with (1) something small, black, and furry, (2) an increased heart rate, (3) negative affect, and (4) one’s own avoidance behavior. With each new observation, these implicit expectations can be adjusted, leading to changes in behavior. For example, after being exposed to a spider for a lengthy period of time and experiencing a gradual reduction in arousal, those same explicit beliefs may come to no longer convey the implicit expectations driving increased heart rate or avoidance (i.e., leading the agent’s model to better approximate the true process generating its observations).

In the simulations shown below, we consider the influence of explicit beliefs on learning from experience in the context of spider phobia. More specifically, we simulate learning through exposure that the presence of a spider does not lead to feared consequences, and we examine how this process differs when explicit beliefs favor danger or safety to different degrees. It is important to stress, however, that spider phobia is meant only as one concrete example. The generic model structure is designed to simulate a wide range of cases in which cognitive restructuring and exposure are used (either separately or together) to reduce maladaptive avoidance behavior. For example, if applied to social phobia we might replace approaching/avoiding a spider with approaching/avoiding a party, and we might replace expectations of serious harm with expectations of social rejection (etc.).

**Figure 2:**
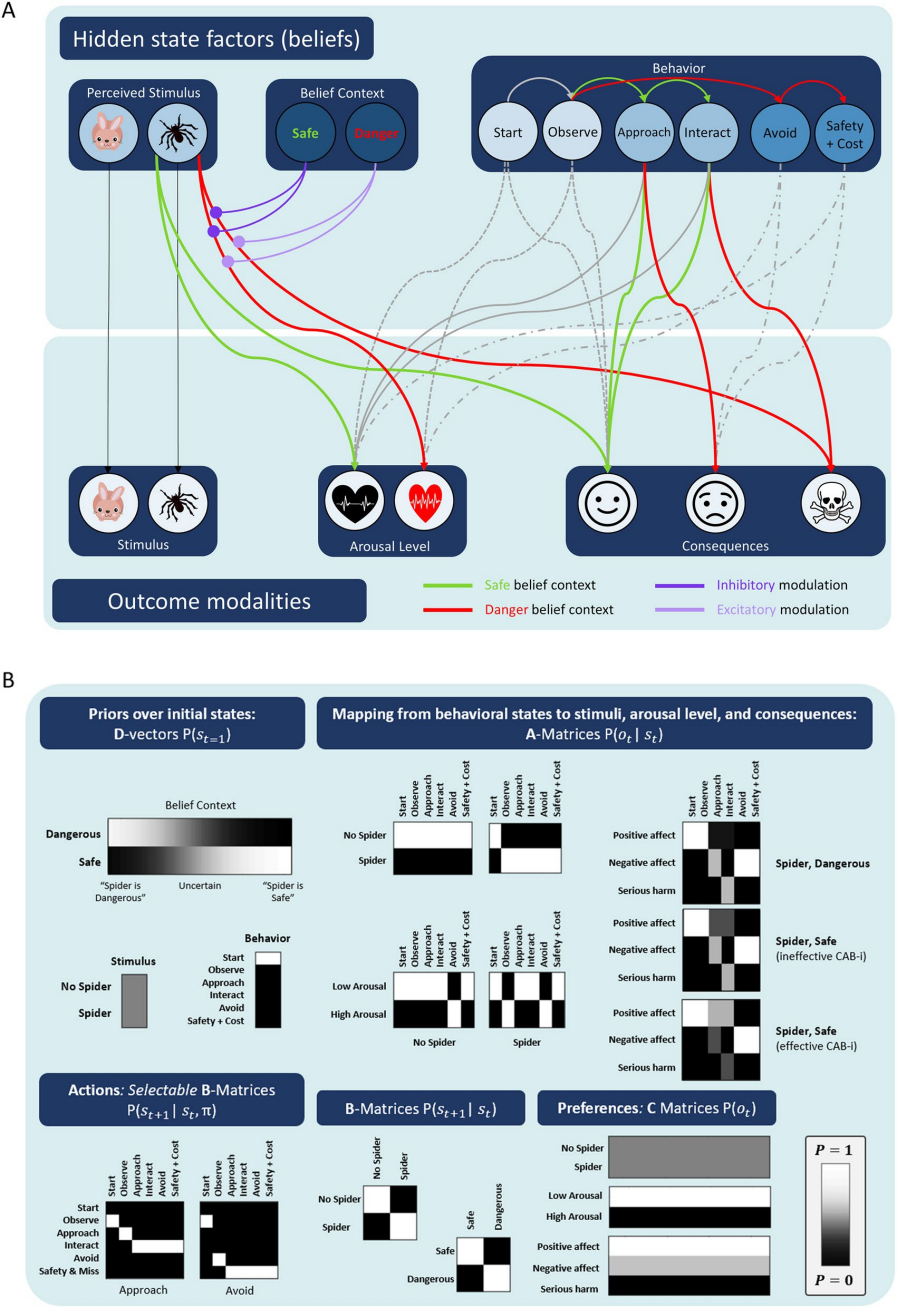
Outline of the generative model. (**A**) Graphical depiction of the generative model, displaying the patient’s beliefs about how different combinations of hidden states generate different combinations of outcomes, as well as how behavior states can transition over time. Red arrows indicate transitions and outcomes generated under “danger” beliefs, while green arrows indicate those under “safe” beliefs (as well as associated avoidance vs. approach transitions, respectively). All other arrows are not dependent on safety/danger beliefs. (**B**) Formal depiction of the model in terms of specification of **A**, **B**, and **C** matrices, and *D* vectors (see text for details and Supplementary Materials: Appendix 1 for associated equations). In these grayscale depictions, lighter values approach 1 and darker values approach 0. CAB-I = cognition-affect-behavior interactions; Safety + Cost = reaching safety along with opportunity costs that promote negative affect. Note that the identity matrix (within the **A** matrices) mapping behavioral states to observed behavioral outcomes is omitted for clarity in both panels.

Specifying a generative model (a model with states that generate/predict outcomes) within the active inference framework requires specification of several vectors and matrices. See Supplementary Materials: Appendix 1 for a detailed mathematical description. The specific generative model we constructed (see Figs. 1 and 2) contained three types of hidden states ($s$):

- Perceived stimulus (no spider, spider).
- Explicit safety beliefs about the spider (dangerous, safe).
- Behavioral states. Behavioral states formally included a “start” state, an “observe stimulus” state, and two behavioral state *trajectories* (i.e., 4 states total: “approach” → “interact” with the stimulus (spider); or “avoid” → “safety + cost,” a state reflecting the fact that avoidance has the cost of missing out on a desired activity or opportunity).

Prior beliefs ($D$ vectors) specified that the agent always started in the “start” state and had equally strong expectations for the presence or absence of the spider. Prior expectations for explicit safety beliefs were manipulated to include probabilistic beliefs about danger vs. safety with different precisions [*danger* 1-*danger*]. Here, high “precision” means that the agent assigns a high subjective probability to one hidden state and a low subjective probability to others (i.e., a probability distribution with a high inverse variance). At one extreme, a “before cognitive restructuring” simulation could be specified as a fully precise belief that “the spider is dangerous” (*danger* = 1). At the other extreme, an “after effective cognitive restructuring” simulation could be specified as a fully precise belief that “the spider is safe” (*danger* = 0). Between these two extremes were a continuum of probability distributions with varying degrees of uncertainty favoring danger vs. safety (e.g., *danger* = 0.9 to 0.1). Note that, for our purposes here, the “safety/danger” beliefs stand in for the more complex patterns of descriptions and appraisals that would be present in a real clinical scenario (e.g., “The spider should not be here and it is awful that it is here”). This simplification was sufficient for our purposes, as our focus was on belief updating processes under different interventions. However, our model could be extended to include more complex appraisals if this was necessary for a different research question.

For our specific purposes, we specified transition maps ($\bf \mathrm{B}$ matrices) such that neither perceptual stimulus states (presence of a spider) nor explicit belief states (danger vs. safety) could change during a single trial (i.e., only after each trial, see below). That is, these were identity matrices. Only behavioral states were under the control of the agent (i.e., choice-dependent $\bf \mathrm{B}$ matrices), with two allowable transition sequences (policies, π) over the 4 time points in each trial: (1) the “approach” policy transitioned *start → observe → approach → interact*; (2) the “avoid” policy transitioned *start → observe → avoid → safety* + *cost*.

There were four outcome modalities ($o$):

- Observed stimulus: no spider, spider (i.e., standing in for sensory input from which the presence or absence of a spider would be inferred).
- Arousal level: low, high (i.e., standing in for ascending signals from the body, such as those indicating high vs. low heart rate).
- Action consequences: positive affect (e.g., feeling happy or content at a social activity), negative affect (e.g., feeling discontent due to the high opportunity cost of avoidance), and serious harm (e.g., being bitten by the spider).
- Observed actions (i.e., allowing the agent to observe its own behavior).

Thus, the state → outcome likelihood maps (the $\bf \mathrm{A}$ matrices) specified:

- An identity mapping from behavioral states to observed actions and from perceived stimuli (i.e., the inferred presence/absence of a spider) to observed stimuli (i.e., sensory input conveying features of a spider vs. no spider; except during the initial “start” state before stimulus presentation, which always mapped to “no spider” observations). These identity mappings entailed that the agent always knew with certainty what actions it performed and whether or not the spider was present.
- First observing a spider always generated brief surprise-like high arousal, that entering the “avoid” state generated high arousal and negative affect (e.g., as when running away), and that all other states generated low arousal (e.g., as when calmly approaching a safe spider in the absence of negative affect, or after successfully avoiding a threat and reaching safety).
- The “safety + cost” state always generated feeling negative affect, reflecting opportunity costs (e.g., leaving a situation one was looking forward to) and other ego-dystonic aspects of avoidance.
- The “approach” and “interact” states led to feeling negative affect and serious harm (respectively) under explicit beliefs that the spider is dangerous, but generated feeling positive affect under the belief that the spider is safe.

In essence, when combined with allowable policies, the likelihood mapping provided the simulated patient with beliefs such that (1) if they thought the spider was dangerous they could choose to run away (which also entails the brain’s “decision” to increase arousal to meet the expected metabolic demands of that behavior), and (2) if they thought the spider was safe they could choose to calmly approach it (which also entails the brain’s “decision” to decrease arousal to meet the low expected metabolic demands of that behavior). For our purposes, we treat the generation of negative affect and high arousal during avoidance (here cast as outcomes) as jointly synonymous with a “negative affective response,” but note that other work in the active inference framework has further highlighted the way changes in pleasant/unpleasant states (orthogonal to arousal) may emerge based on unexpected decreases/increases in expected free energy upon new observations^11^. Note also that the exact details of the situation and the semantic description of what we simulate here are not essential, which allows the model to generalize to other situations with minimal changes. It is only the abstract structure/dynamics of the model that determine our simulation results below (e.g., where the outcome of approaching a safe stimulus leads to relatively greater well-being than avoidance, but that avoidance leads to relatively greater well-being than approaching a truly harmful stimulus).

It’s important to highlight the way these state → outcome mappings capture CAB interactions in the cognitive triangle. First, the explicit safety/danger beliefs (cognition) influence affective responses (valence/arousal) and approach/avoidance (behavior). Second, observations of low/high arousal and positive/negative affect both provide evidence supporting either safety or danger beliefs, respectively (e.g., all else being equal, sustained high arousal reinforces beliefs in danger). Third, low/high arousal, positive/negative affect, and approach/avoidance are mutually supportive, as low arousal and positive affect are more consistent with calmly approaching a situation than running away to avoid it. Thus, all types of CAB interactions are captured. The exact structure of these mappings can also be arranged to capture different levels of efficacy for CAB interactions. Specifically, and as also depicted in Fig. 2B, a CAB interaction strength parameter (CAB-i) could be defined within the portion of the likelihood mapping ($\bf \mathrm{A}$ matrix) encoding the probability, under different explicit beliefs, of positive affect, negative affect, and serious harm when choosing to approach and interact with the spider:

$$P\left({o}_{action-consequences}|{s}_{approach\to interact}, {s}_{safe},{s}_{spider}\right)= \left[\begin{array}{cc}CABi& CABi\\ 1-CABi& 0\\ 0& 1-CABi\end{array}\right]$$

From left to right in this matrix, the columns correspond to the “approach” and “interact” states; from top to bottom, the rows correspond to the “positive affect”, “negative affect”, and “serious harm” outcomes. Thus, the higher the CAB-i value, the greater the implicit expectation that positive affect would be experienced if one chose to “approach” the spider while believing it was safe.

The preference-for-outcomes map (formally, the $\bf \mathrm{C}$ matrix containing the prior log-probabilities of observations) was set as follows: the aversion to “serious harm” was set to $-12$, “high arousal” and “negative affect” were both assigned a value of $-1$, and all other observations were assigned a value of $0$. These values are normalized to form a proper probability distribution using a softmax function, and then the values in this distribution are converted into log-probabilities. For details on parameter specification choices, see Supplementary Materials: Appendix 2.

## Results

### Foundational simulations

**Figure 3:**
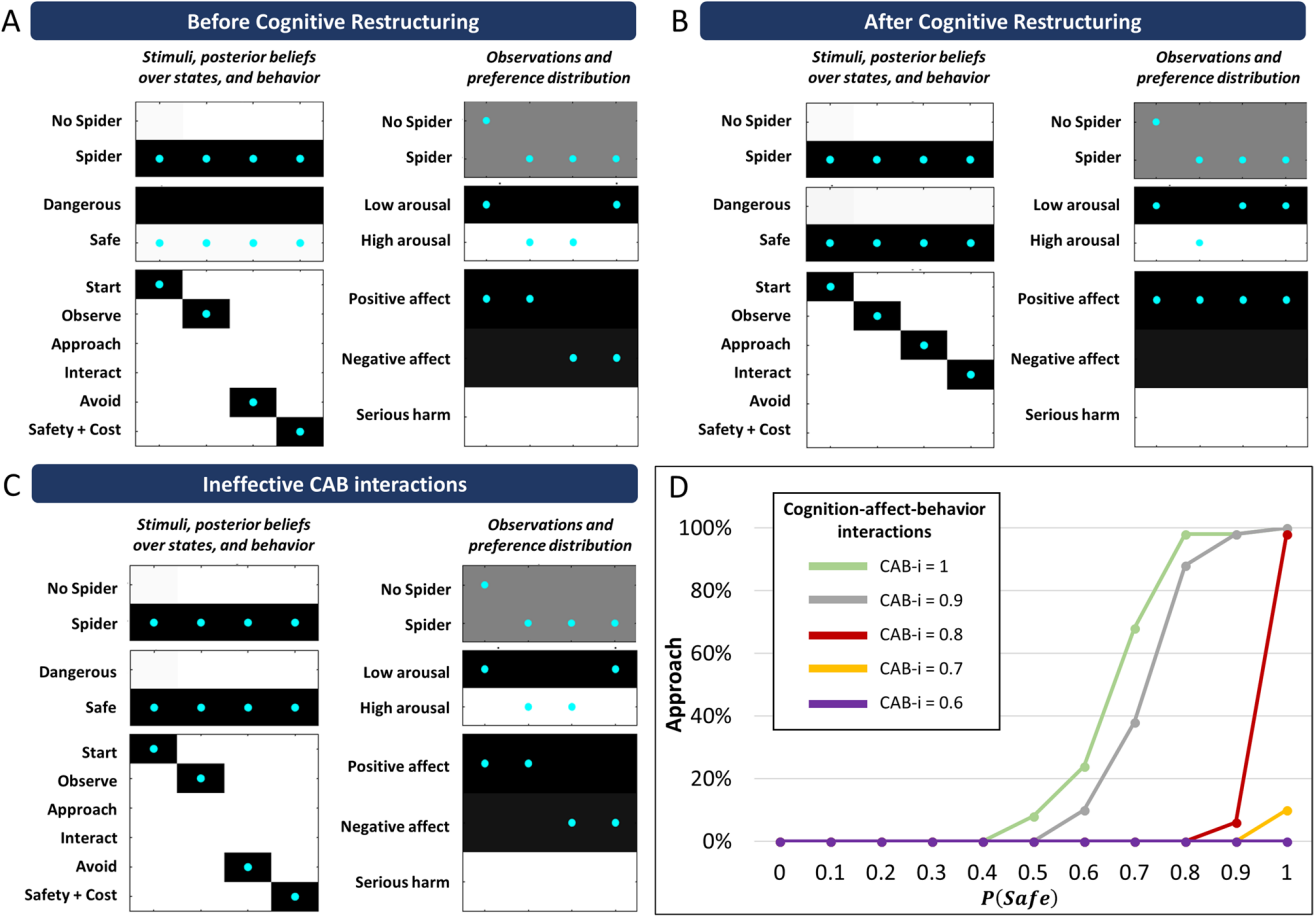
Illustration of single-trial simulations. Within the left column of each panel, darker values indicate stronger beliefs favoring the associated states, whereas darker values in the right column of each panel indicate stronger preferences for the associated outcomes. Cyan dots indicate the true states and outcomes. See main text for interpretation. (**A**) Simulation under precise danger beliefs (*danger* = 1). (**B**) Simulation under precise safety beliefs (*danger* = 0). (**C**) Simulation under precise safety beliefs with ineffective CAB interactions. (**D**) The percentage of simulated trials (out of 100) in which the simulated patient chose to approach the spider, given different explicit beliefs about the probability that the spider was safe. Each colored line illustrates the behavioral curve under CAB interactions of different efficacies (higher = more efficient). CAB-i = parameter encoding the efficacy of cognition-affect-behavior interactions; Safety + Cost = reaching safety along with opportunity costs that promote negative affect.

This setup afforded simulations of approach and avoidance behavior under different safety/danger beliefs, effective vs. ineffective CAB interactions, and before vs. after simulated exposure therapy. In our first, foundational simulation, we show that “before cognitive restructuring” (i.e., precise danger beliefs; *danger* = 1), the simulated patient chooses to avoid when presented with the spider (Fig. 3A, bottom left), leading to a high arousal state until reaching safety (Fig. 3A, middle right). In contrast, “after cognitive restructuring,” which we operationalize here as the induction of precise safety beliefs (*danger* = 0), the patient chooses to approach, leading to a faster transition back to a low arousal state after initial surprise (Fig. 3B).

### Ineffective CAB interactions

These first two simulations assumed that CAB interactions were highly effective. However, as discussed above, there are many cases in which—despite a patient consciously endorsing an explicit belief that a problem stimulus/situation (e.g., spider) is safe—unpleasant high arousal and avoidance responses persist. In our model, ineffective CAB interaction of this type can be reproduced by lowering the influence of explicit safety beliefs on the mappings from the spider percept to arousal levels and expected behavioral consequences within the $\bf \mathrm{A}$ matrix (see depiction of the $\bf \mathrm{A}$ matrix in Fig. 2B associated with “ineffective CAB-i”). Effectively, this brings the implicit expectations conveyed under safety beliefs to become more like those conveyed by danger beliefs—rendering negative affective responses relatively insensitive to explicit beliefs. In the simulation depicted in Fig. 3C it can be seen that, despite an explicit belief in safety, a sustained unpleasant, high-arousal response is still generated and the agent remains motivated to avoid. Note, however, that in this type of Bayesian architecture, all interactions between states and observations are bidirectional. This entails that weak CAB interactions also hinder explicit belief updating from observed outcomes, whereas strong CAB interactions facilitate explicit belief updating. Consequently, ineffective CAB interactions imply two sources of burden: prolonged physiological stress, and hindered learning (i.e., explicit belief updating) through experience.

Building on the foundational simulations above, to more fully characterize the model’s choice behavior we ran repeated simulations across a range of possible prior expectations and CAB interaction strengths (Fig. 3D). As can be seen, the probability of approach increased with stronger safety beliefs, but the influence of safety beliefs on behavior was sharply attenuated when CAB interactions were reduced. However, under strong explicit danger beliefs and strong CAB interactions (green line), the agent continued to avoid over 100 trials if permitted. Thus, even though our model promoted a plausible amount of randomness in behavior (i.e., due to the moderate inverse temperature parameter [decision noise] value; see Supplementary Materials: Appendix 2), this was insufficient to allow safety learning. Continued avoidance under strong explicit danger beliefs therefore prevented the agent from making observations that would update both implicit and explicit beliefs in a manner that would promote approach behavior. This is consistent with associative (e.g., actor-critic) accounts wherein an agent’s behavior similarly prevents observations that would extinguish avoidance^22^. However, our model finesses this understanding by incorporating a much fuller range of influences, analogous to thoughts and emotions; it also further clarifies the role of uncertainty, which is crucial in anxiety-related conditions. One important point is that, despite preventing corrective observations, a patient’s avoidance behavior in these simulations is internally rational (i.e., it is optimal given beliefs in the model). This highlights the important (and in some cases necessary) role of a therapist in guiding individuals to explore the outcomes of approach behavior within maladaptively feared situations.

### Effects of explicit beliefs on learning through exposure

**Figure 4:**
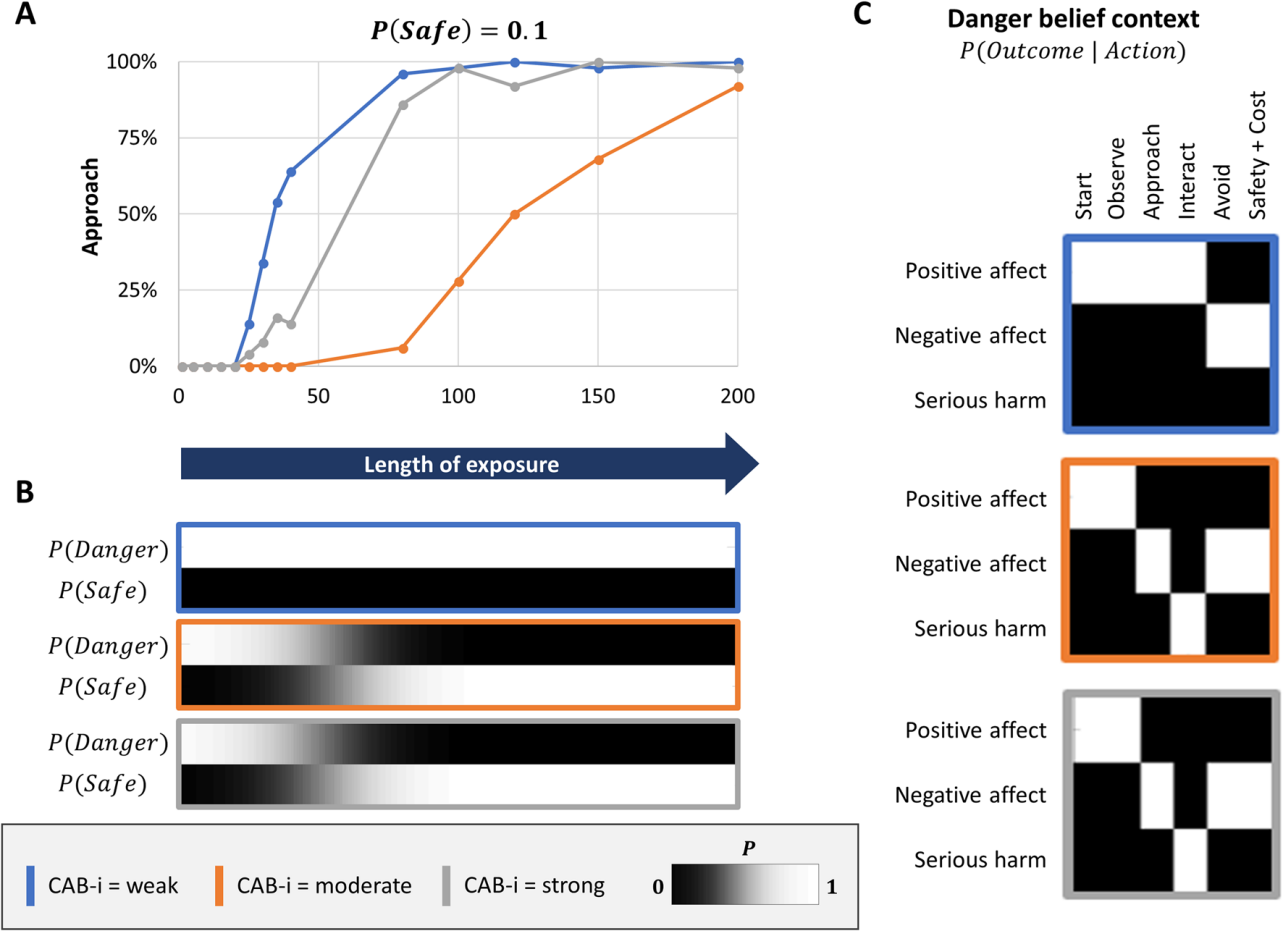
Exposure therapy under strong explicit danger beliefs (i.e., as in the absence of cognitive restructuring; $P(safe) = .1$) and different CAB interaction strengths. Approach behavior increased with length of exposure (panel** A**), but the underlying mechanisms differed depending on CAB interaction strengths. When CAB interactions were moderate (CAB-i = .5, orange outline) or strong (CAB-i = .9, grey outline), behavior change was the result of updates in explicit beliefs (panel** B**)—leaving implicit beliefs (panel** C**) unchanged and creating a vulnerability to the re-emergence of negative affect and avoidance responses in other contexts. Under weak CAB interactions (CAB-i = .1, blue outline), explicit danger beliefs remained unchanged (e.g., the simulated patient continued to have the automatic thought “this spider is dangerous”; panel** B**), but implicit beliefs (panel** C**) slowly adjusted such that avoidant affective responses attenuated over time—and were independent of changes in explicit beliefs. Learning was slower under moderate CAB interactions (.5, orange outline; panel** A**). As discussed in the main text, this is because the formal matrix structure in this case can also be interpreted as the patient being unsure *that a safe context is possible* (i.e., the hidden state for the possibility of another context started out making completely uninformative predictions)—thus, the patient needed to first infer the presence of a meaningfully distinct context. CAB-i = parameter encoding the efficacy of cognition-affect-behavior interactions; Safety + Cost = reaching safety along with opportunity costs that promote negative affect.

**Figure 5:**
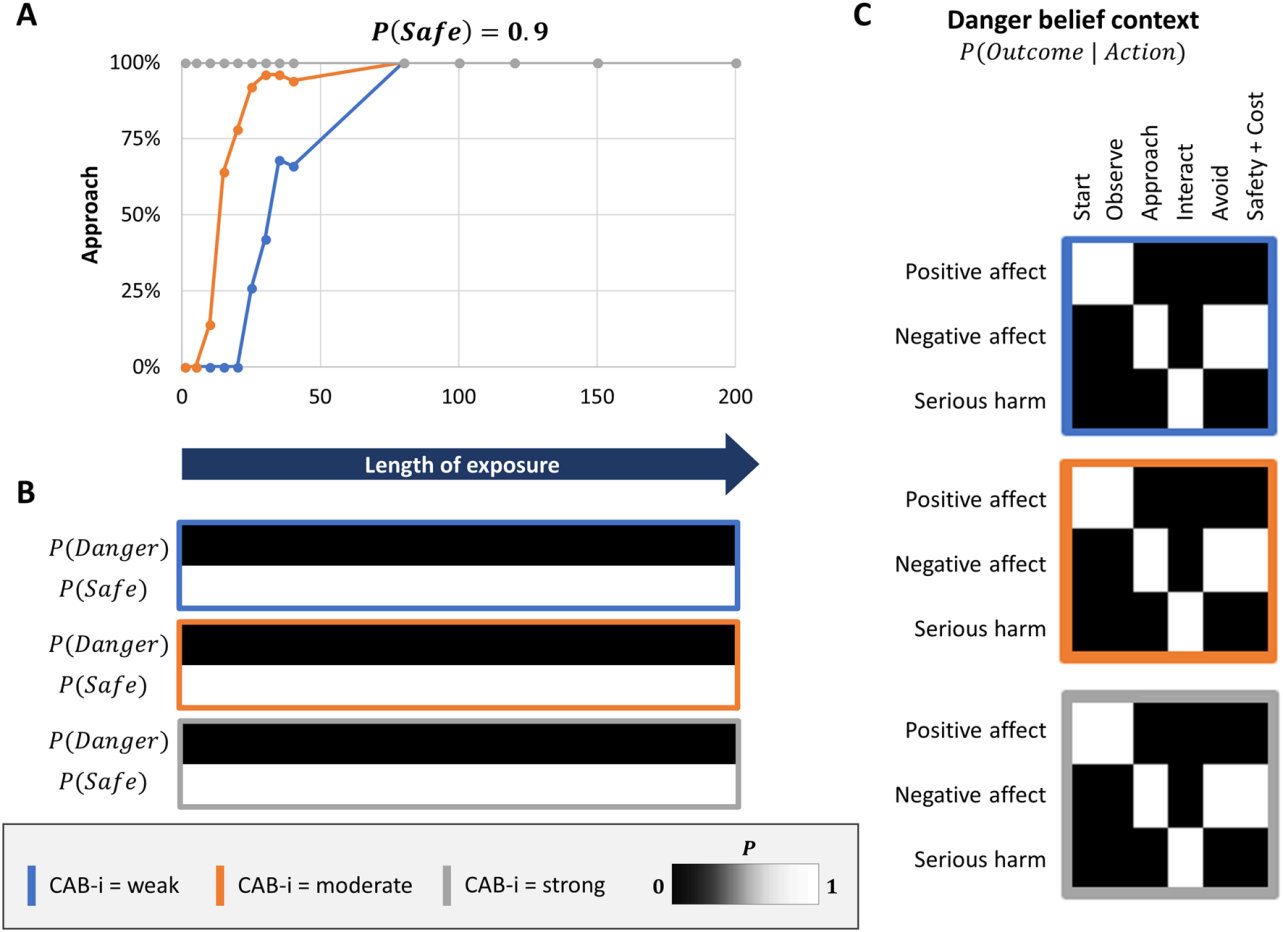
Exposure therapy under strong explicit safety beliefs (i.e., such as after cognitive restructuring; $P(safe) = .9$) and different CAB interaction strengths. When CAB interactions were strong (grey outline), approach behavior was present immediately (panel** A**). Under moderate or weak CAB interactions (orange or blue outline), behavior change occured rapidly. Implicit beliefs (panel** C**) associated with explicit beliefs in danger (panel** B**) remained unchanged, creating a vulnerability to the re-emergence of negative affective responses and avoidance behavior in other contexts (i.e., upon the return of thoughts of danger). CAB-i = parameter encoding the efficacy of cognition-affect-behavior interactions; Safety + Cost = reaching safety along with opportunity costs that promote negative affect.

**Figure 6:**
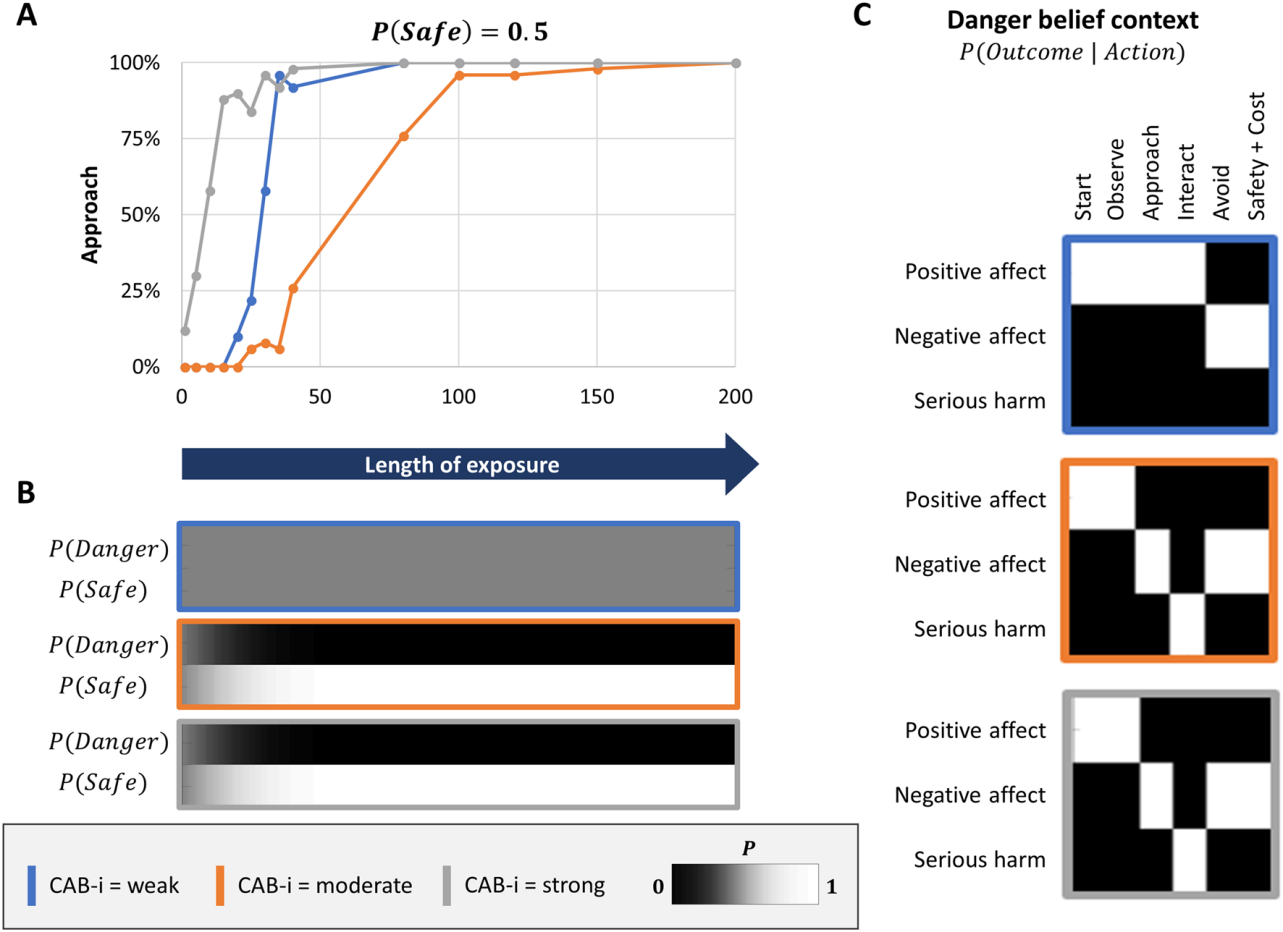
Exposure therapy under uncertain explicit beliefs (i.e., as could also be facilitated by cognitive restructuring; $P(safe) = .5$) and different CAB interaction strengths. Approach behavior increased with length of exposure at a faster rate than under strong danger beliefs, but the underlying mechanisms differed depending on CAB interaction strengths (panel** A**). When CAB interactions were moderate or strong (orange or grey outline), behavior change was the result of updates in explicit beliefs (panel** B**)—again leaving implicit beliefs unchanged (panel** C**) and creating a vulnerability to the re-emergence of avoidance if thoughts of danger were to return. Under weak CAB interactions (blue outline), explicit danger beliefs remained uncertain (e.g., the simulated patient continued to have the thought “this spider may or may not be dangerous”; panel** B**), but implicit beliefs (panel** C**) slowly adjusted such that avoidant affective responses attenuated over time—and were independent of changes in explicit beliefs (panel** B**). Learning was slower under moderate CAB interactions (panel** A**). As mentioned in the legend for Fig. 4, this was because the formal matrix structure can in this case also be interpreted as the patient being unsure *that a safe context is possible* (i.e., the hidden state for the possibility of another context started out making completely uninformative predictions)—and must therefore first infer the presence of a meaningfully distinct context. CAB-i = parameter encoding the efficacy of cognition-affect-behavior interactions; Safety + Cost = reaching safety along with opportunity costs that promote negative affect.

Subsequently, our primary simulations examined learning dynamics under synthetic exposure therapy and its potential interactions with cognitive restructuring (Figs. 4, 5, 6). Exposure was implemented by simulating 200 trials in which we prevented the agent from choosing avoidance behavior (formally, this was carried out by specifying a highly precise prior expectation for selecting the approach policy in a vector $E$). Please note that this should *not* be thought of as 200 exposure *sessions*, but instead as an index of the amount of time the simulated patient is continuously exposed to the spider (i.e., length of exposure). After various exposure lengths, we allowed the agent to freely select the approach or avoid policy and assessed the degree to which learning from exposure had altered behavior. The percentage of approach decisions displayed was over 50 repeated simulations.

To better understand the underlying learning mechanisms, we also investigated how the agent updated explicit beliefs ($D$) about safety, and implicit beliefs ($\bf \mathrm{A}$) about the outcomes of approach vs. avoidance. This allowed us to assess whether exposure learning remained specific to one explicit belief or instead generalized across different explicit beliefs (e.g., whether avoidance behavior would re-emerge if thoughts of danger returned).

#### Danger beliefs

The results of exposure therapy when the agent began with strong explicit danger beliefs, $P(safe) = .1$, are shown in Fig. 4. This resembles a situation in which exposure is implemented without first addressing explicit cognitive beliefs about danger in therapy. As depicted in blue, an exposure length of roughly 100 time points (formally modeled as trials) was necessary to produce consistent approach behavior when CAB interactions were weak (CAB-i = 0.1). Here the agent did not update explicit beliefs in safety vs. danger. Instead, behavioral change was explained by updates to implicit (associative) beliefs about the outcomes of approach behavior. “Approach” and “interact” were now implicitly expected to produce feeling positive affect. In other words, the original implicit belief that approach behavior would lead to ‘serious harm’ was effectively overwritten. As depicted in gray, an exposure length of roughly 100 time points was also necessary to produce consistent approach behavior when CAB interactions were strong (CAB-i = 0.9). In this case, however, the agent did update explicit beliefs in favor of safety (i.e. the spider was now seen as safe). Implicit beliefs about outcomes under the other possible explicit belief (i.e., under the thought that the spider was dangerous) remained unchanged—still resulting in unpleasant high arousal and avoidance. This means that, if the agent were to again believe it was in the presence of a dangerous spider, negative affective responses and avoidance behavior would be expected to return. In other words, despite each of these two simulations resulting in identical approach behavior, *the computational mechanisms underlying behavioral change were different*—and they predict different long-term behavioral outcomes. Specifically, when behavior change is due to updating implicit beliefs, return of negative affect and avoidance (i.e., clinical relapse) would be less likely because the reduced avoidance motivation isn’t dependent on explicit belief states. In contrast, relapse would be more likely when behavior change is due to updating explicit beliefs, because avoidance motivation remains dependent on state inference (i.e., avoidance motivation could reappear if automatic thoughts of danger returned). As depicted in orange, results in the case of moderate CAB interaction strengths (CAB-i = 0.5) were similar to the strong CAB-i case—with the exception that a greater length of exposure (roughly 200 time points) was necessary to produce consistent approach behavior (we return to this below).

#### Safety beliefs

The results of exposure therapy when the agent began with a strong explicit safety belief, $P(safe) = .9$, are shown in Fig. 5. This resembles a situation in which a patient has already gained strong explicit beliefs that the spider is safe as a result of cognitive restructuring. In this case, explicit beliefs were not meaningfully altered by exposure. Implicit beliefs about action outcomes were also only updated under the explicit belief in safety, and therefore did not generalize to beliefs that a spider is dangerous. CAB interactions at low and moderate levels increased the length of exposure needed to produce reliable approach behavior. In summary, when explicit beliefs already strongly favored safety, exposure could produce behavior change in the context of weaker CAB interactions, but it hindered the possibility that these changes in affective/behavioral responses would generalize to other contexts (i.e., they would re-emerge if thoughts of danger returned).

#### Uncertain beliefs

The results of exposure therapy when the agent began with maximal uncertainty in explicit beliefs, $P(safe) = .5$, are shown in Fig. 6. This resembles a situation in which, instead of leading to confident safety beliefs, cognitive therapy promoted a general reduction in explicit belief precision (e.g., helping a patient to allow alternative interpretations of a situation to exist in their mind simultaneously, as in leading approaches within CBT^2^). As depicted in blue in Fig. 6, an exposure length of less than 50 time points was necessary to produce consistent approach behavior when CAB interactions were weak (CAB-i = 0.1). Here the agent remained uncertain in its explicit beliefs, and therefore updated its implicit (associative) beliefs about the outcomes of approach behavior equally under both possible explicit beliefs. Approach behavior therefore generalized, which could be thought of as automatic affective responses becoming independent of continued thoughts of danger. As depicted in orange and gray, when CAB interactions were moderate (CAB-i = 0.5) or strong (CAB-i = 0.9), the agent did gain strong explicit safety beliefs. Implicit beliefs about the outcomes of actions were only updated under explicit beliefs in safety, preventing generalization (i.e., allowing the possibility of re-emergence of avoidance when thoughts of danger return). Moderate CAB interactions led to slower behavioral change.

In the simulations just described, it is worth briefly returning to the finding that, under what we have called “moderate CAB interactions”, behavioral change tended to occur more slowly than at weaker or stronger CAB interactions. This result can be better understood when recognizing that, formally, this parameterization can also be thought of as encoding maximum uncertainty about whether there *is* more than one possible explicit belief. In other words, under this parameterization the agent has precise expectations about what it will observe with a dangerous spider, but has no specific expectations about what other patterns of outcomes might be observed. Thus, the agent’s model allows for the possibility that a different belief might be possible (i.e., with a different action-outcome mapping)—but it must infer that this maximally imprecise mapping actually provides a better explanation for new observations than the informative mapping it already has for its danger belief. Behavioral change is therefore slower in this context because the agent must first infer the existence of a “safe” context and then begin to learn the new action-outcome relationships in that context (formally, this corresponds to a type of “structure learning” problem^23^).

These simulations make an interesting prediction. Specifically, they suggest that, instead of aiming to adjust explicit beliefs toward safety in cognitive therapy (e.g., aiming to elicit responses from patients such as “I know the spider won’t hurt me during exposure”), when combining cognitive and behavioral (exposure) interventions, it may be more effective to only induce uncertainty in explicit beliefs (e.g., only aim to elicit responses from patients such as “It’s possible the spider could hurt me but it’s also possible that it won’t”). Somewhat counterintuitively, the model suggests that, under this type of uncertainty, it may also be better to try to *decrease* the influence of cognitive states on affect/behavior—for example, by helping a patient to distance themselves from their thoughts (e.g., to see them as “just thoughts”, to “not buy into them”, etc.^2,7^). The specific prediction is that the effects of exposure therapy may generalize better to other contexts when patients’ explicit beliefs remain highly uncertain (low-precision) and when the influence of automatic thoughts is itself attenuated via metacognitive (e.g., mindful acceptance-based) interventions.

## Discussion

In this paper, we studied an agent before and after simulated cognitive-behavioral therapy (CBT^1,2^), using spider phobia as a concrete example. We captured learning during behavioral (exposure-based) interventions as well as how they may interact with the effects of cognitive restructuring (e.g., modifying confidence in automatic thoughts that a spider is safe or dangerous). In addition, we demonstrated how the effects of explicit beliefs on behavior can be moderated by how effectively cognitive levels of processing interact with the implicit associations that maintain ingrained avoidance responses to feared stimuli (“cognitive-affective-behavioral” [CAB] interactions). Implicit beliefs in this sense specified, for example, that a perceived dangerous stimulus generated increased arousal and feelings of negative affect, or that a safe stimulus generated low arousal and positive affect. Our model reproduced the CAB interactions in the cognitive triangle of CBT, such that, when such interactions are strong, cognition, affect, and behavior each mutually influenced each other to promote consistency (e.g., negative affect promoted thoughts of danger, high arousal, and avoidance motivation). This naturally flowed from the model architecture that we used, wherein the probability of each type of hidden state (i.e., Bayesian belief) was inferred given the state probability estimates in other parts of the model (via interaction with observed outcomes); all model elements were thus automatically driven toward maximizing overall belief consistency. When these CAB interactions were weak, negative affective/behavioral responses could persist despite more adaptive conscious thoughts. These weak interactions also allowed behavior change during exposure to occur even when negative automatic thoughts persisted.

We first confirmed the hypothesis that avoidance behavior in a freely behaving agent prevented the observations necessary to learn more adaptive approach behaviors. That is, our model produced plausible patient behavior before therapy—reacting with high arousal and always avoiding the spider, while holding strong beliefs that it was dangerous to approach it. This replicated the thoughts, unpleasant visceral responses, and avoidant behaviors especially prominent in patients with anxiety disorders, as in our spider phobia example. Consequently, the simulated patient missed the chance to interact with the spider and experience neutral, benign outcomes, and so failed to update (either implicit or explicit) beliefs on their own, regardless of the number of opportunities to approach.

We further found that exposure therapy was effective at reducing avoidance behavior in all simulations; this included cases of stronger and weaker CAB interactions, as well as cases where a patient explicitly believed spiders in the current context were either dangerous (“I will be seriously harmed”) or safe (i.e., after cognitive restructuring). This was also true when explicit beliefs were highly uncertain (“maybe I’ll be safe, maybe I’ll be seriously harmed”). However, one major insight offered by the model was that, while behavior change occurred in all cases, the underlying computational learning processes differed between cases. That is, what was learned was not always the same.

Without cognitive restructuring, and if CAB interactions were strong, behavioral change was primarily due to updating explicit beliefs. The simulated patient came to believe the current spider was safe. Implicit associative beliefs remained unchanged, such that if thoughts about danger returned then spiders would again evoke arousal, negative affect, and avoidance. This could be the case, for example, if one saw a different kind of spider or the same spider in a different context. We observed a similar result if simulated cognitive restructuring had engendered strong explicit safety beliefs prior to exposure. That is, while avoidance was reduced, implicit associations were not affected. Both situations therefore did not change the simulated patient’s predispositions to react to thoughts about spiders being dangerous, rendering the agent vulnerable to the re-emergence of avoidance.

There were only two cases, both when CAB interactions were weak, in which implicit beliefs were altered during exposure and thus reduced vulnerability to the re-emergence of avoidance. The first case was when the patient entered exposure with strong explicit beliefs that the spider was dangerous. The second was if cognitive restructuring had only induced strong uncertainty about whether the spider was dangerous (note that avoidance also reduced more quickly in this latter case). In these situations, because explicit beliefs about the context remained unchanged, behavior change was primarily due to “over-writing” the implicit associative beliefs linking spiders to unpleasant arousal and avoidance. We interpret this as affective responses being rendered less sensitive to automatic thoughts. The potential downside to this, however, was that, due to the necessary bidirectional nature of Bayesian message passing in active inference models, weak CAB interactions also prevented observations from updating explicit beliefs. In general, since the associative beliefs were overwritten, this entails they would not spontaneously return. Some existing empirical work speaks to this result. For example, work by Gershman and colleagues (e.g.,^16,19^) has formulated the spontaneous return of avoidance responses after fear extinction in terms of inferring safe vs. dangerous latent causes, which is analogous to the safe vs. dangerous explicit beliefs in our model. This formulation is consistent with empirical work^14^ demonstrating a reduced return of fear responses when exposure involves gradual (as opposed to abrupt) reductions in the observed relationship between conditioned and unconditioned stimuli—presumably promoting the inference that the original latent cause has gradually changed its causal properties (i.e., as opposed to promoting the inference that a new latent cause is present). Our results build on these by generating the prediction that this reduction in the return of fear responses will be facilitated by uncertainty induction and helping patients distance the relationship between their thoughts and actions. This could complement other common explanations of fear reinstatement, which have focused on whether expectation violations are experienced within a single context vs. across many contexts. As reviewed by Craske and colleagues^24^, violation of expectations is an important component of exposure therapy, while more commonly discussed reductions of anxiety and arousal are poorer predictors of therapeutic success. However, if expectation violations only occur in a single context, the new non-threat associations that are learned may not generalize beyond that context. Thus, it has been suggested that exposure should be optimized to facilitate the formation of non-threat associations across multiple contexts. The threat vs. non-threat associations in this prior work map well onto the implicit beliefs in our model, while the different (typically environmental) contexts in this prior work play a similar functional role as the explicit belief contexts (thoughts of danger vs. safety) in our model. Thus, our results offer the additional element of explicit belief context as a potentially relevant factor for which generalization may also be important.

In this context, it is important to again highlight that, while we have used spider phobia as an illustrative example, the structure of the model is general and much more widely applicable. For example, it could equally apply to avoiding public spaces in agoraphobia, avoiding parties in social anxiety disorder, and avoiding relationships due to a fear of rejection, among many others. The main difference would just be what the relevant explicit vs. implicit beliefs are (e.g., likelihood of a panic attack in public, social rejection, or abandonment). The therapeutic goal in such disorders is not necessarily the eradication of anxiety. Instead, the patient is to be empowered to deal with any analogous, anxiogenic contexts they might encounter in the future. Patients learn to differentiate, observe, and intervene within all three domains of the cognitive triangle. Specific skills are acquired to question one’s own thought processes and form new helpful inferences; for example, by using the ABC-model in CBT, where an *Activating event* triggers *Beliefs* and their emotional *Consequences*^25^. Emotion regulation techniques including mindful acceptance, as well as “behavioral experiments” (collaboratively designing new actions and examining their effects, often to safely test the patient’s specific negative expectations) complement this. They allow the patient to have more choice in how to react to future events. When mastered, such cognitive-behavioral and affect regulation skills unlock self-efficacy towards any potential context, feared or not. The individual can feel content, even if the context may be unsafe. Consequently, a dreaded context can be approached by the individual, because arousal and affective states are now under control.

However, while the coexistence of thoughts of danger with approach behavior can be useful for patients to achieve goals that they otherwise wouldn’t, it also has some problematic aspects in the account we have given so far. Specifically, the cognitive-behavioral approach, and cognitive restructuring specifically, does not simply concern itself with the content of thoughts, but also with their rational inference and helpful function. Given certain contexts, re-experiencing the thought “this situation is dangerous” may be an appropriate inference, such as when visiting a location *where dangerous spiders actually live*—and thus avoidance may be an appropriate response. This highlights how therapy should also avoid overgeneralization. Our model should therefore be seen as only addressing maladaptive “danger beliefs” when they reflect a poor or otherwise unhelpful model of the world (i.e., when beliefs are unwarranted in context, as may occur during catastrophizing, anxious rumination, or depressive negative automatic thoughts). In actual cases, a patient will also need to learn to discriminate the situations in which unpleasant avoidance responses are and are not helpful.

A related issue worth addressing is that, while we have formally explored the mechanisms of updating prior expectations over states (explicit beliefs) and updating likelihood mappings (implicit beliefs), active inference models offer other, complementary computational mechanisms that could produce approach behavior, with potentially distinct psychological implications. A primary example would be reducing a patient’s confidence in the expected outcomes of actions (i.e., reducing the value of the expected policy precision term $\gamma$; see Supplementary Materials: Appendix 1). Reducing policy precision can lead to more random “exploratory” behavior, which could facilitate observations of safe outcomes. This is analogous to the random exploration that gradually leads to extinction in older, temporal-difference models of fear conditioning^22^. Another example would be how repeated approach decisions can result in an “approach habit”; in active inference, this involves an individual building up prior expectations over policies as a result of repeated approach decisions ($i.e., p\left(\pi \right),E$). In this case, a “habitual approach” pattern would develop independent of other beliefs. A further interesting effect, as observed in recent active inference models of medication adherence^26^, involves the interaction between $\gamma$ and $E$—in which less confidence in belief-based action selection (lower $\gamma$) can also increase the strength of the influence of habits ($E$) on behavior.

A further consideration is the potential role of learning rates. In some computational models, learning rates are dynamic; they increase when environments are believed to be volatile (i.e., when the relationships between actions and outcomes are unstable), and decrease when environments are believed to be stable (i.e., because unexpected observations are more likely due to randomness in a stable environment, and beliefs should therefore not change drastically from a single unexpected observation). A dynamic learning rate was not formally included in our model, but if this were included it would represent yet another way in which uncertainty induction (about action-outcome relationships) in therapy might facilitate behavioral change (i.e., by increasing learning rate). Consideration of these other mechanisms illustrates how, aside from the benefits of inducing uncertainty in beliefs about danger, fostering uncertainty in action outcomes (as well as learning automatic approach habits) might have additional benefits and speed improvement. Successful therapy most plausibly targets many of these mutually reinforcing mechanisms simultaneously. That said, it is important to consider both the possible positive and negative consequences of such mechanisms. For example, it may also be important for learning rate to subsequently decrease after behavioral change has occurred—highlighting the need to also reinforce confidence in what has been learned during therapy (e.g., solidifying learning by helping a patient maintain attention to, and linguistically process, unexpected outcomes during exposure^24,27^). These considerations are also highlighted by results of previous empirical studies that have found differences in learning rates within anxious populations. For example, one study found evidence that anxious individuals had more difficulty adjusting learning rates in unstable environments^28^, and another found evidence of elevated learning rates generally (i.e., representing a general belief in the unpredictability of the world^29^). Thus, sustained elevated learning rates will not always be adaptive, and therapists may need to help patients adjust their learning rates dynamically during exposure. This represents an important future direction for extending the model we describe here.

Based on the neural process theory associated with active inference^21,30^, our model may also suggest important hypotheses about the distinct mechanistic basis of explicit vs. implicit belief updating—highlighting how distinct neural mechanisms can produce the same phenotypic behavior change. First, explicit, reportable beliefs are expected to correspond to current patterns of firing rates in specific neuronal populations that play the role of representing probability distributions over different possible beliefs (here, plausible brain regions include those associated with more abstract state representations, such as ventromedial prefrontal cortex and hippocampus, among others^31–33^). These transient firing patterns are most plausibly what inform self-reportable thoughts. Changing these thoughts can occur relatively quickly, as they are the result of inference and are updated after single observations (e.g., verbally reasoning with a therapist). In contrast, implicit associative beliefs are expected to correspond to particular patterns of synaptic connection strengths that convey the expected outcomes associated with specific percepts and actions (e.g., expecting harm when approaching a spider). These connection strengths are adjusted more slowly, as they reflect the outcome statistics across many past experiences and may require many new observations before changing substantially (plausibly associated with interactions between cortical representations and the amygdala and other subcortical visceromotor control regions^34^). Thus, cognitive and behavioral interventions are suggested to target specific, distinct neural substrates. (Note: our brief discussion of inducing action uncertainty and habits [prior expectations over policies] also further suggests distinct dopaminergic and lateral basal ganglia mechanisms, respectively^35–38^).

It’s important to highlight the limited scope of the model. First, as mentioned above, we did not distinguish rational vs. irrational explicit danger beliefs. Second, we did not directly address the mechanisms associated with CAB interactions, which could include both implementation breakdown (e.g., ineffective axonal message passing along axons between prefrontal and subcortical regions), higher metacognitive influences (e.g., mindfulness “turning down” these interactions adaptively), or relatively “hard-wired” responses (e.g., fearful avoidance of skydiving despite knowing it’s safer than other common daily activities like driving). More complex models could explicitly consider these elements. Additional insights might be gained if the model were extended to include beliefs about additional hidden states and CAB interactions. This could also draw on existing clinically-motivated models that propose a larger number of interacting cognitive subsystems^39^ and multiple information processing routes at both the cognitive and neural level for generating affective and behavioral responses^40,41^. Elements of our current model (e.g., explicit vs. implicit beliefs) have rough analogues in these other proposals (e.g., propositional/schematic vs. associative representations^40^; or higher vs. lower “roads” from sensory to subcortical brain circuits^41^), but our model would require additional components to more fully capture them. That said, our model can offer insights about the computational mechanisms that may underlie the processes described in those models.

Yet another important topic not addressed is intolerance of uncertainty, which is an important individual difference in affective disorders^42^. Active inference agents have inherent drives to seek information, but with parameters that can control the relative drive to seek information vs. reward^43^. Agents with high tolerance for uncertainty would be expected to choose to approach or avoid despite uncertainty about safety vs. danger—which, depending on the level of fear of serious harm—could either solidify avoidance or facilitate approach (but also facilitate generalization since uncertainty would be more likely to persist). In contrast, low tolerance for uncertainty would drive the agent to seek information until it was confident in safety vs. danger before deciding to approach vs. avoid, which would hinder generalization in our model. However, if partial approach was a means of information-seeking, this could also facilitate willingness to engage in exposure. That said, it’s important to highlight that, while these consequences are expected, we cannot be confident about these outcomes until explicit simulations are performed. It will be an important future direction to extend our model so as to allow such simulations.

It was also beyond the scope of the paper to explicitly simulate some other mechanisms mentioned above, such as habit learning, changing confidence in action outcomes, dynamic learning rates, influences of prior expectations, and selective attention biases. These each represent important directions for future work and could make further empirical predictions. It is also worth noting that this type of modelling assumes approximately optimal decision-making under a given set of beliefs—such that maladaptive behavior follows from inaccurate beliefs^44^. Models with this type of internal optimality can be used even in the presence of substantial neuroanatomical lesions^45–47^, but simulating potential clinical effects of lesions was also beyond our scope here. Finally, as in any modelling project of this type, parameter choices must be made/found to produce plausible behavior. Empirical work will be necessary to fit these parameters to real world behavior to confirm the validity of these choices.

## Conclusion

Our model offers a number of important advantages and insights. It formalizes therapeutic mechanisms and allows quantitative modelling of interactions between cognition, affect, arousal, and behavior, which captures phenomena central to assumptions of several prominent psychotherapeutic approaches. It demonstrates how distinct influences of cognitive restructuring and the strength of CAB interactions (plausibly modified by metacognition-focused interventions such as mindful acceptance training^7,48–50^) can interact with the mechanisms and outcomes of exposure therapy. It also makes interesting predictions about how uncertainty induction and efforts to reduce CAB interactions using metacognitive interventions could lead to more lasting and generalizable effects of exposure. This therefore represents an important step in advancing the nascent field of computational psychotherapy.

### Software note

Our model simulations can be implemented using standard routines (here spm_MDP_VB_X.m) available as Matlab code in the latest version of SPM academic software: http://www.fil.ion.ucl.ac.uk/spm/. The simulations in this paper can be reproduced (and customized) via running the Matlab code included here in the supplementary material (CBT_model.m). This code can also be found at: https://github.com/rssmith33/Simulating_Cognitive_Behavioral_Therapy.

## Supplementary Information

Supplementary material.

Supplementary Appendix 1-2.
