# Supplementary material for: Simulating the computational mechanisms of cognitive and behavioral psychotherapeutic interventions: insights from active inference
Source: Sci Rep. 2021 May 12;11:10128. doi: 10.1038/s41598-021-89047-0 (PMC8115057; doi:10.1038/s41598-021-89047-0)
Supplement: Supplementary file 2 — Supplementary Appendix 1-2. [file 41598_2021_89047_MOESM2_ESM.pdf]

Supplementary Materials

Simulating the computational mechanisms of Cognitive and Behavioral Psychotherapeutic Interventions: Insights from active inference

Ryan Smith[1], Michael Moutoussis[2,3]*, Edda Bilek[2]*

[1]Laureate Institute for Brain Research, Tulsa, OK, USA

[2]Wellcome Centre for Human Neuroimaging, Institute of Neurology, University College London

[3]The Max Planck – University College London Centre for Computational Psychiatry and Ageing

*authors contributed equally and are designated as joint senior authors

Appendix 1: A primer on active inference

We employ the active inference framework and the general structure of the partially observable Markov Decision Process (POMDP) model. For a more full derivation and explication of the mathematics, the reader is referred to [44]. POMDP models within the active inference framework require specification of specific types of random variables, vectors, and matrices, as well as specific starting (prior) beliefs that influence the behavior of interactions between these model elements. For a given time in trial (*t*), vectors for random variables include observations (o_t^m) that can be grouped into separate modalities (*m*), probability distributions (a.k.a. beliefs) over hidden states (s_t^f) that can be divided into different factors (*f*), and probability distributions over policies (π), where each policy is a sequence of actions an individual can choose. These models also include a vector (D^f; one per state factor) that specifies initial ("prior") beliefs over states ($p(s_{t=1}^f)$), and a matrix ($\mathbf{C}^m$; one per observation modality) that specifies which observations are more/less pleasant than others for each observation modality at each time point. Formally, $\mathbf{C}^m$ is a fixed (log-)prior bias over expected observations, $lnp(o_t^m)$). Matrices relating these variables

include a “likelihood” matrix ($\mathbf{A}^m$; one per outcome modality) that specifies observations expect under hidden states ($p(o_t^m|s_t^f)$) and a matrix ($\mathbf{B}_{\pi,t}^f$; one per state factor) that specifies prior beliefs about how hidden states evolve over time and their dependence on chosen policies ($p(s_t^f|s_{t-1}^f,\pi)$). Updated (“posterior”) beliefs over states after each new observation ($p(s_t^f|o_t^m)$)), here denoted as $\bar{s}$**,** are based on approximately optimal (Bayesian) integration of state priors and likelihoods.

When *t* = 1:

$$\bar{s}_{\pi,t} = \sigma(\frac{1}{2}(lnD + ln\mathbf{B}_{\pi,t} \cdot s_{\pi,t+1}) + ln\mathbf{A} \cdot o_t)$$

When t > 1:

$$\bar{s}_{\pi,t} = \sigma(\frac{1}{2}(ln\mathbf{B}_{\pi,t-1}s_{\pi,t-1} + \ ln\mathbf{B}_{\pi,t} \cdot s_{\pi,t+1}) + ln\mathbf{A} \cdot o_t)$$

These equations indicate that updating beliefs after a new observation involves a kind of compromise between prior expectations ($D\ or\ \mathbf{B}$) and what is most consistent with the new observation (based on $\mathbf{A}\ and\ o$). Note that, by convention in the active inference literature, the dot operator (·) here denotes transposed matrix multiplication, and σ denotes the softmax (normalized exponential) function.

The probability of selecting one policy over others is based on minimizing statistical quantities (originally borrowed from physics) referred to as variational free energy (F_π) and expected free energy (G_π). F_π is a measure of the accuracy of the observations predicted under the state transitions specified by each policy, but also takes into account how much beliefs need to change to maximize accuracy (where a policy is “better” if beliefs need to change less). G_π scores the

probability of observing preferred outcomes under each policy, but also takes into account how much information can be gained (a policy is better if it maximizes both the probability of preferred observations and information gain). Agents also hold a prior expectation over policies $p(\pi)$denoted by E, which encodes habit-like beliefs that some policies are more likely to be selected over others. They are habit-like in the sense that they are not actively inferred, but instead accumulate over experience through having been selected more often in the past. The overall probability distribution over policies, from which actions are sampled at each time point, is then:

$$\pi = \sigma(lnE - \gamma G - F)$$

Where:

$$F_{\pi} = \sum_{t} s_{\pi,t} \cdot (ln\, s_{\pi,t} - \frac{1}{2}(ln\mathbf{B}_{\pi,t-1} s_{\pi,t-1} + \; ln\mathbf{B}_{\pi,t} \cdot s_{\pi,t+1}) - \mathbf{A} \cdot o_{t})$$

$$G_{\pi} = \sum_{t} (o_{\pi,t} \cdot (ln\, o_{\pi,t} - ln\mathbf{C}) - \; diag(\mathbf{A} \cdot ln\mathbf{A}) \cdot s_{\pi,t} \; - \mathbf{A}s_{\pi,t} \cdot \mathbf{W}s_{\pi,t})$$

The novelty term **W** reflects how much beliefs about **A** are expected to change under each policy (defined below in relation to learning), which means that policies are more likely to be chosen if they are expected to maximize information gain about the relationship between states and observations. The expected policy precision parameter γ denotes confidence in the contribution of expected free energy to policy selection, where this level of confidence can be updated with each new observation (based on changes in expected free energy). When this value is low, an agent becomes less sensitive to differences in G_{π}, due to a lack of confidence in their model of the world. In this case, policy selection becomes more random and more influenced by habits (E). We do not focus on γ in our explicit simulations below (setting it to a value of 1, such that it

has no influence on policy selection), but we do return to it in the discussion. To produce plausible amounts of variability in choice behavior, such that decisions are sensitive to the probabilities assigned to possible actions at each timepoint in a trial, we do set a fixed inverse temperature parameter (α) to a value of 4 during action selection, where $P(Action|\alpha) = \sigma(\alpha \times lnP(Action|\pi)$.

Finally, learning within this class of models is based on updating prior beliefs over the categorical distributions in the vectors/matrices described above. These updates take place through the accumulation of Dirichlet concentration parameters, which parametrize these distributions. In our case, we simulate learning Dirichlet priors (d) and ($\boldsymbol{a}$) for explicit danger/safety beliefs (D) and implicit beliefs about the outcomes of actions ($\mathbf{A}$), respectively. The form of these updates are as follows:

$$d_{trial} = d_{trial-1} + s_t$$

$$\boldsymbol{a} = \boldsymbol{a}_{trial-1} + \sum_t o_t \otimes s_t$$

Where $\otimes$ denotes the cross product. This translates to the agent simply adding (proportions of) counts to each distribution, based on the number of times the agent believes it has occupied a given state; and the number of times it has observed a specific outcome when it believes it occupied a given state. As mentioned above, policy selection is also influenced by information-seeking, such that policies are preferred if they are expected to increase confidence in state-outcome mappings during learning. This is accomplished through the novelty term shown above, defined as:

$$\mathbf{W} := \frac{1}{2}(\boldsymbol{a}^{\odot(-1)} - \boldsymbol{a}_{sums}^{\odot(-1)})$$

Note that the $:=$ symbol just means that two things are defined to be equivalent; and the $\odot$ symbol indicates the element-wise power (i.e., separately raising each element in a matrix to the power of some number). The term $\boldsymbol{a}_{sums}$ is a matrix of the same size as $\boldsymbol{a}$ where each entry within a column corresponds to the sum of the values of the associated column in $\boldsymbol{a}$.

Appendix 2: Model Parameter Specification

Prior beliefs (D) in our model specified that the agent always started in the "start" state, [1 0 0 0 0 0]', and had equally strong expectations for the presence or absence of the spider, [.5 .5]. Prior expectations for explicit safety beliefs were manipulated to include different probabilistic beliefs about danger vs. safety [*danger* 1-*danger*]. At one extreme, a "before cognitive restructuring" simulation could be specified as a fully precise belief that "the spider is dangerous" (*danger* = 1). At the other extreme, an "after effective cognitive restructuring" simulation could be specified as a fully precise belief that "the spider is safe" (*danger* = 0). Between these two extremes were a continuum of probability distributions with varying degrees of uncertainty favoring danger vs. safety (e.g., *danger* = .9 - .1).

As in the main text, full characterization of the model's choice behavior was evaluated through repeated simulations across a range of possible prior expectations and CAB interaction strengths. Here, however, for parameter selection we further assessed the influences of different preference strengths, by examining how the resulting behavioral curves were influenced by different "harm aversion" levels (formally, the magnitude of the preference against the "serious harm" outcome in **C**). **Figure S1** shows these results, where each graph depicts the percentage of approach (vs. avoidance) decisions across 100 repeated simulations under different harm aversion levels

$(lnp(o) = -10, -11, or -12$; i.e., low, medium, high aversion), and each colored line indicates a different CAB interaction strength. In the main text, we used the harm aversion level of $lnp(o) = -12$, which reproduced a behavior pattern demonstrating plausibly high avoidance levels. Specifically, the agent here required roughly 80% confidence in safety before choosing to approach and risk the possibility of the "serious harm" outcome. As stated in the main text, preferences in **C** for other observations were as follows: "high arousal" and "negative affect" were both assigned a value of $lnp(o) = -1$; all other observations were assigned a value of $lnp(o) = 0$.

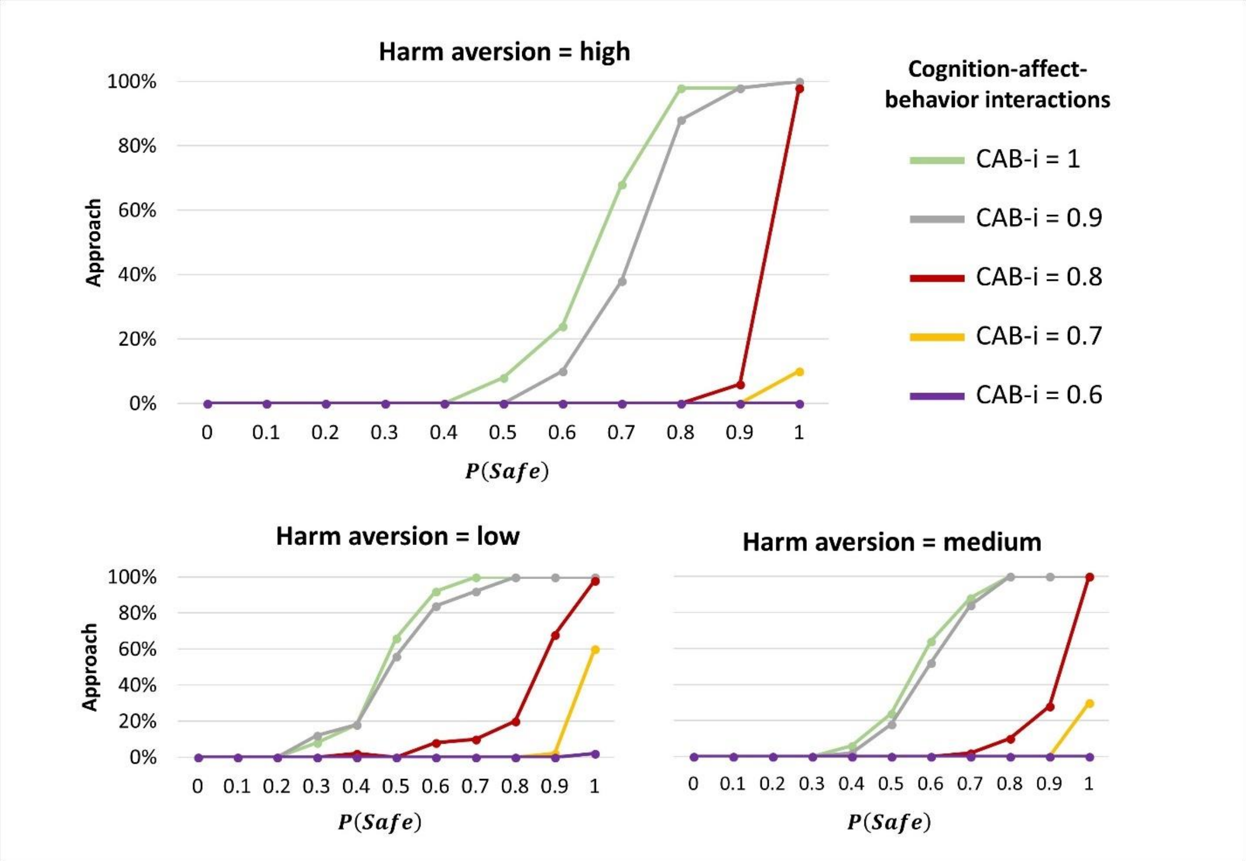

Figure S1. Model behavior. Each graph illustrates the percentage of simulated trials (out of 100) in which the simulated patient chose to approach the spider, given different explicit beliefs about the probability that the spider was safe. Each colored line illustrates the behavioral curve under CAB interactions of different efficacies (higher = more efficient). Each panel shows the resulting curves under different levels of 'harm aversion' (i.e., the magnitude of the preference against the "serious harm" outcome; see text for exact values). As expected, approach behavior increased with stronger safety beliefs, but only with

sufficiently strong CAB interactions. A high harm aversion level of $lnp(o) = -12$ produced strong (clinically relevant) avoidance behavior and was used for subsequent simulations. CAB-i values = cognition-affect-behavior interaction strengths.

To simulate confident initial prior beliefs in danger and expected observations under approach vs. avoidance, concentration parameters in d for these beliefs were multiplied by 50 (e.g., [1 0] = [50 0]; [.5 .5] = [25 25]), and concentration parameters in $\boldsymbol{a}$ for mappings to affect and serious harm were multiplied by 5. Effects of other settings for these parameters were explored to ensure results were not strongly dependent on particular values. We found, as expected, that decreasing d or $\boldsymbol{a}$ magnitudes caused learning (and behavior change) to occur at an effectively faster rate during the synthetic exposure therapy simulations shown in the main text – but this did not affect the qualitative nature of our findings. Adjusting values of $\boldsymbol{a}$ also modulated the influence of the magnitude of harm aversion encoded in $\mathbf{C}$. However, by finding an appropriate value for $\mathbf{C}$, plausible approach/avoidance behavior could be produced under a wide range of possible a values. Thus, this also did not meaningfully affect the qualitative pattern of results we report below. To prevent learning with respect to other mappings irrelevant to our simulations, all other concentration parameters in d or $\boldsymbol{a}$ were multiplied by a very high value of 128.
